# Neurobiological correlates of successful deep brain stimulation in a mouse model of high trait affect

**DOI:** 10.1186/2050-6511-13-S1-A44

**Published:** 2012-09-17

**Authors:** Claudia Schmuckermair, Stefano Gaburro, Anupam Sah, Rainer Landgraf, Simone B Sartori, Nicolas Singewald

**Affiliations:** 1 Department of Pharmacology and Toxicology, Leopold-Franzens-University of Innsbruck, 6020 Innsbruck, Austria; 2 Department of Physiology, Westfälische Wilhelms-University Münster, 48149 Münster, Germany; 3 Max Planck Institute of Psychiatry, 80804 Munich, Germany

## Background

Recent evidence suggests that high-frequency deep brain stimulation of the nucleus accumbens (NAcb-DBS) may represent a novel therapeutic strategy for individuals suffering from treatment-resistant depression although the underlying mechanism of action remains largely unknown. Using a unique psychopathological mouse model of enhanced depression- and anxiety-like behavior (HAB) we investigated behavioral and neurobiological effects of NAcb-DBS.

## Methods

HAB mice underwent either chronic treatment with different selective serotonin reuptake inhibitors (SSRIs) or stereotactic surgery to implant DBS electrodes into the NAcb. NAcb-DBS was applied for 1 h daily for seven consecutive days (130 Hz, 100 µA, 60 µs pulse width) and sham-stimulated animals were used as controls. Anxiety- and depression-related behaviors were assessed using established tests with predictive anxiolytic or antidepressant validity. Furthermore, the effects of NAcb-DBS on challenge-induced immediate early gene expression and hippocampal neurogenesis were investigated.

## Results

Chronic SSRI treatment did not alter the enhanced depression-like behavior of HAB mice, while repeated, but not single, NAcb-DBS induced robust antidepressant and anxiolytic responses. Interestingly, NAcb-DBS did not affect behavior in normal depression/anxiety animals (NAB), suggesting a preferential effect of NAcb-DBS on pathophysiologically deranged systems. Antidepressant-like effects of NAcb-DBS were associated with normalization of challenge-induced dentate gyrus hypoactivity and modulation of neuronal activity in various brain regions implicated in stress and depression. Furthermore, NAcb-DBS enhanced the blunted hippocampal neurogenesis in HABs.

## Conclusions

Taken together we show that the normalization of pathophysiologically enhanced depression-like behavior by repeated NAcb-DBS was associated with normalization of aberrant brain activity and rescue of impaired adult neurogenesis, indicating that DBS affects gene expression as well as neuronal plasticity in a defined, mood-associated network. Finally, it is suggested that SSRI-insensitive HAB mice represent a clinically relevant model for elucidating the neurobiological correlates underlying the observed behavioral effects of NAcb-DBS.

